# Marginal Quality and Wear of Bulk-Fill Composites: Differences Between Dentitions

**DOI:** 10.3290/j.jad.c_1865

**Published:** 2025-02-07

**Authors:** Maria Hofmann, Emma Wolf, Susanne Lücker, Roland Frankenberger, Bernd Wöstmann, Norbert Krämer

**Affiliations:** a Maria Hofmann Assistant Professor, Department of Pediatric Dentistry, Medical Center for Dentistry, University Medical Center Giessen and Marburg, Campus Giessen, Schlangenzahl 14, 35392 Giessen, Germany. Supervised the experiments, data analysis, wrote the manuscript.; b Emma Wolf Doctoral Student, Department of Pediatric Dentistry, Medical Center for Dentistry, University Medical Center Giessen and Marburg, Campus Giessen, Schlangenzahl 14, 35392 Giessen, Germany. Performed the experiments.; c Susanne Lücker Research Fellow, Department of Pediatric Dentistry, Medical Center for Dentistry, University Medical Center Giessen and Marburg, Campus Giessen, Schlangenzahl 14, 35392 Giessen, Germany. Supervised the experiments.; d Roland Frankenberger Professor and Chair, Department of Operative Dentistry, Endodontics, and Pediatric Dentistry, University of Marburg, Medical Center for Dentistry, University Medical Center Giessen and Marburg, Campus Marburg, Georg-Voigt-Strasse 3, 35039 Marburg, Germany. Study concept, proofread the manuscript.; e Bernd Wöstmann Professor and Chair, Department of Prostodontics, Medical Center for Dentistry, University Medical Center Giessen and Marburg, Campus Giessen, Schlangenzahl 14, 35392 Giessen, Germany. Supervised thermomechanical loading and wear analysis.; f Norbert Krämer Professor and Chair, Department of Pediatric Dentistry, Medical Center for Dentistry, University Medical Center Giessen and Marburg, Campus Giessen, Schlangenzahl 14, 35392 Giessen, Germany. Study idea, supervised the experiments, proofread the manuscript.

**Keywords:** bulk-fill composite resins, marginal quality, permanent teeth, primary teeth, restoration materials, wear

## Abstract

**Purpose:**

The aim of this study was to evaluate the marginal quality and wear of bulk-fill composite resins (BFs) for Class-II restorations of primary and permanent molars in comparison to a conventionally layered composite resin (RC) and to compare the results of the two dentitions.

**Materials and Methods:**

Eighty (40 primary and 40 permanent) extracted molars received standardized Class-II cavity preparations and were restored with either one of two flowable BFs, one of two high viscous BFs, or a composite resin (RC). Thermomechanical loading (TML; 2,500 cycles +5°C/+55°C; 100,000 cycles, 50N, 1.67Hz) followed. A quantitative marginal analysis using SEM images and a profilometric quantification of two-body wear were carried out using replicas. ANOVA, Kruskal–Wallis, Mann–Whitney U, and Wilcoxon signed-rank tests were used for statistical analysis (P < 0.05).

**Results:**

For both dentitions, a significant reduction of perfect margins was observed after TML (P < 0.02). For the primary dentition, the flowable BFs showed significantly less perfect margins than all high viscous materials (P < 0.005). For the permanent dentition, RC showed significantly fewer gaps than the flowable BFs (P < 0.04). Regarding wear, within the dentitions, no significant differences could be computed between groups with regard to the maximum height loss (P < 0.05).

**Conclusion:**

All of the investigated bulk-fill composite resins showed satisfactory *in-vitro* results for both tested parameters in primary and permanent teeth, with a superiority of the high-viscosity materials in terms of marginal quality.

Recently, bulk-fill composite resins (BFs) are used more frequently in the permanent as well as in the primary dentition. In comparison to conventional composite resins (RCs), BFs offer an easier and time-saving application procedure due to their increased depth of cure.^8^ This is an improvement compared to conventional layering techniques with a maximum thickness of 2 mm per increment, and it can be of special interest for patients that need short dental sessions, ie, children.

Light-curing BFs, where curing (time) is controllable, can be applied in increments of 4–5 mm.^8^ The increased depth of cure (DOC) of the BFs can be achieved by multiple modifications of the material characteristics. Some bulk-fill composites contain UDMA as a monomer, which has a smaller molecular size compared to other conventional monomers and therefore a higher concentration of double bindings for the same volume. This leads to a higher network density and tighter network formation in the polymer.^48^ Bulk-fill composite resins, as a further factor causing an increased DOC, can contain modified initiator systems that, in contrast to the conventional camphorquinone initiator system, lead to the formation of more free radicals, which could start the radical polymerization reaction.^40^ Furthermore, the fillers in most bulk-fill composites are significantly larger than in conventional composites, which reduces the interface between the fillers and the matrix, which increases translucency and in turn favors a higher DOC.^24^ The increased DOC of BFs can further be achieved through reduced filler content and accompanying less light diffusion.^24^ However, a reduced filler content is further related to reduced mechanical properties, such as hardness and flexural strength.^35^ Furthermore, a higher translucency is associated with compromised esthetics, which results in BFs being less favored in the visible area. More and more BFs now are being offered in tooth shades and sometimes even with a chameleon effect in the color matching. Moreover, BFs can be divided into high-viscosity (sculptable) and low-viscosity (flowable) materials^8^ according to their consistency, which is also related to the filler content.^2^

Class-II restorations, which are located in the posterior region of the dental arch, bear the chewing load.^33^ Therefore, also adequate wear resistance is an important factor for a successful overall performance of the restoration.^34,50^ To prevent wear, the application of so-called capping layers of highly viscous RC over the bulk-fill material is often recommended for low-viscous BFs with their lower filler content. Since the enamel of primary teeth is softer than permanent teeth enamel due to its lower mineral content,^25^ it is assumed that the two dental hard tissues have different wear resistances. Consequently, BF restorations in the primary dentition are often not capped.

Furthermore, the capping layers consisting of an RC should also be used to improve esthetics of BF restorations.^8^ Recently, most manufacturers also offer their BFs in non-universal/tooth-colored shades to make bulk-fill composite restorations more esthetically pleasing.

In addition to wear resistance, marginal seal is of special importance for the success of bonded restorations. Microleakage and marginal gap formation can lead to the development of secondary caries,^26^ ie, a carious lesion beneath the filling margin. Marginal gaps can occur as a result of polymerization shrinkage of the filling material if the resulting polymerization shrinkage stress within the material exceeds the strength of the adhesive bond,^12^ or due to aging processes, which potentially lead to the degradation of the tooth-composite interface.^6^ In the primary dentition as well as in the permanent dentition, recurrent caries, and marginal defects are among the predominant failure reasons for posterior composite resin restorations.^9,28^

In the permanent dentition, a successful clinical performance could be demonstrated for BFs with follow-up times of up to ten years, being comparable to that of RCs.^5,31^ With regard to the clinical examination of the performance of BFs in primary posterior teeth, there are currently only a few heterogeneous studies available, presenting follow-up periods of a maximum of 24 months.^1,14,32,36,42,43,47^ Furthermore, no studies have been published that compare clinical performance of BFs in primary and permanent teeth.

The aim of this *in-vitro* study was to evaluate the marginal quality and wear of tooth-colored BFs as Class-II restorations of primary and permanent molars compared to the use of an RC. Moreover, it was intended to examine how the two dentitions compared to each other using these two parameters for the respective restorative material tested in each dentition.

## MATERIALS AND METHODS

The methodology used in this study has previously been described by Hofmann et al,^21^ the protocol was approved by the local IRB (ethical committee of the Justus-Liebig-Universität Giessen (file reference 143/09)).

After extraction, disinfection, and storing, 40 primary and 40 permanent molars were randomly assigned to 10 groups (5 groups with permanent and 5 groups with primary teeth; n = 8). After preparation of standardized Class-II box-only cavities (dimensions: coronal-cervical = 2.0–3.0 mm, mesial-distal = 1.5–2.0 mm, buccal-oral = 3.0–3.5 mm) using a diamond-coated cylinder (8835KR.314.010, ISO 806 314 156514 010, length: 4.0 mm, Komet Dental/Gebr. Brasseler & Co., Lemgo, Germany), enamel and dentin were bonded and cavities were filled with different bulk-fill composite resins and one conventional composite resins. For permanent teeth, the etch-and-rinse technique using a 35 % phosphoric acid (DeTrey Conditioner 36, Dentsply Sirona Deutschland, Bensheim, Germany; etching time: 30 s enamel, 15 s dentin), was used prior to the application of the universal adhesive iBond Universal (Kulzer, Hanau, Germany), which was used for all study groups. iBond Universal was applied according to the manufacturer’s instructions (primary teeth: self-etch mode) and was light-cured (10 s) after application.

The examined composite resins and the group categorization are shown in Table 1. BFs were applied using the bulk-fill technique. One conventional composite, which was applied in an oblique incremental technique, was used as a control group for each dentition. Each composite layer was light-cured for 20 s (Bluephase G2, Ivoclar Vivadent, Schaan, Liechtenstein; light intensity: 1,200 mW/cm^2^). The light source was positioned in the immediate vicinity of the filling material from the occlusal direction, and the light output was controlled before the filling process of each test group (CURE RITE VISIBLE CURING LIGHT METER, Dentsply Caulk, Milford, USA: 1783–1877 mW/cm^2^). For flowable materials, the filling process was supported by the use of a matrix (Tofflemire Matrizen 0,05 mm, Art.-Nr. 47673, Pluradent AG & Co. KG, Offenbach, Germany).

**Table 1:** Grouping (n = 8), abbreviations and properties of composite materials under investigation

1 (FUK-1)	3M™ Filtek™ Universal Komposit Z250 (3M Deutschland, Seefeld)	Conventional composite, packable	Primary
2 (FOB-1)	3M™ Filtek™ One Bulk Fill (3M Deutschland, Seefeld)	Bulk-fill composite, packable
3 (SDR-1)	SDR® flow+ (Dentsply DeTrey, Konstanz)	Bulk-fill composite, flowable
4 (TPF-1)	Tetric® PowerFill (Ivoclar Vivadent, Ellwangen (Jagst))	Bulk-fill composite, packable
5 (VBF-1)	Venus® Bulk Flow ONE (Kulzer, Hanau)	Bulk-fill composite, flowable
11 (FUK-2)	3M™ Filtek™ Universal Komposit Z250 (3M Deutschland, Seefeld)	Conventional composite, packable	Permanent
12 (FOB-2)	3M™ Filtek™ One Bulk Fill (3M Deutschland, Seefeld)	Bulk-fill composite, packable
13 (SDR-2)	SDR® flow+ (Dentsply DeTrey, Konstanz)	Bulk-fill composite, flowable
14 (TPF-2)	Tetric® PowerFill (Ivoclar Vivadent, Ellwangen (Jagst))	Bulk-fill composite, packable
15 (VBF-2)	Venus® Bulk Flow ONE (Kulzer, Hanau)	Bulk-fill composite, flowable

After finishing and polishing using different polishing discs and composite polishers (3M™ Sof-Lex™ polishing discs, Article numbers 1981M, 1981F and 1981SF, 3M™ Deutschland, Germany; Identoflex™ composite polisher, ID 5021, ID 5061, ID 5081, Kerr, Biberach, Germany), tooth samples were incubated at 37°C for 28 days (Incubator Typ B20 Heraeus Holding, Hanau, Germany; T1) and were then subjected to thermocycling (TCS 30, Syndicad, Munich, Germany; 2,500 cycles; +5°C/+55°C; T2) as well as a mechanical loading in a chewing simulator (100,000 cycles; antagonist steatite: 6 mm diameter; 50 N; 1.67 Hz; T3). Between each of the steps, silicone impressions were taken for the production of replicas (AlphaDie MF, Schütz Dental, Rosbach, Germany).

**Fig 1:**
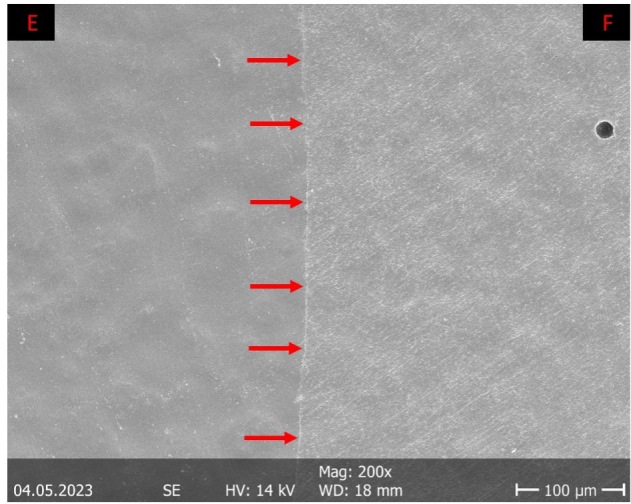
Margin analysis: Exemplary illustration of the evaluation criterion “perfect margin” (scanning electron micrograph of the filling margin at 200 x magnification). Enamel (E) and filling material (F) are flush with each other and lie in one plane (red arrows).

**Fig 2:**
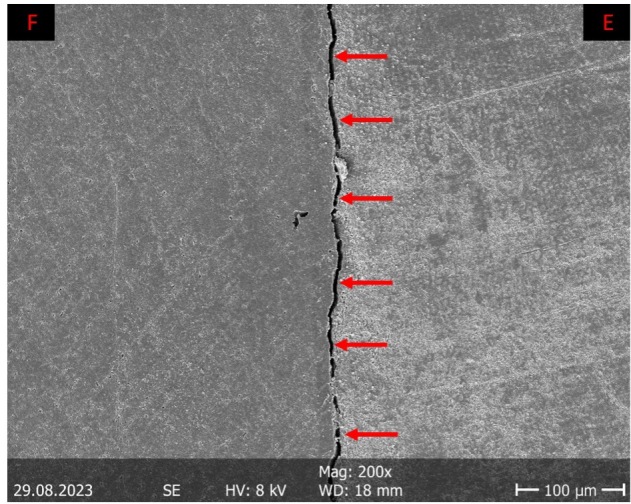
Margin analysis: Exemplary illustration of the evaluation criterion “gap” (scanning electron micrograph of the filling margin at 200 x magnification). There is a visible open margin (appears as a line-shaped shadow; red arrows) between the filling material (F) and the enamel (E), which is accompanied by no significant loss of substance in the filling or the tooth structure.

One part of the replicas (T1, T2) was then used for scanning electron microscopic (= SEM; Amray 1810, Amray, Bedford, MA, USA; 10 kV accelerating voltage; 200× magnification) margin analysis (software tools: (“Fiji is Just Image J” Freeware, Wayne Rasband, National Institutes of Health, Bethesda, MD, USA; “KHK’s Quantigap,” Freeware, KHK). Each section of the proximal filling margin was assigned one out of seven (“perfect margin”/“overhang”/“positive step formation”/“negative step formation”/“gap”/“non-judgeable”/“fracture”) criteria. The proportion of the respective evaluation criterion in relation to the total length of the respective filling margin was analyzed quantitatively. This proportion was expressed as a percentage [%]. An exemplary illustration of the criteria “perfect margin” and “gap” can be found in Figures 1 and 2.

**Fig 3:**
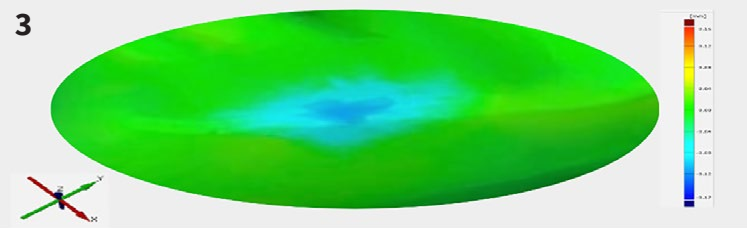
Wear analysis: representation of maximum height loss (structured light scanner; ATOS CORE 45 & GOM Inspect 2020); “Region of interest”: dark blue center with light blue border.

The other part of the replicas was scanned with a structured light scanner (ATOS Core 45, measuring volume: 45 × 30 × 25 mm, working distance: 170 mm, point spacing: 0.02 mm; software program: ATOS Prof 2018; both GOM, Braunschweig, Germany) and matched (GOM Inspect 2020, GOM; superimposition technique, T2 versus T3) for the measurement of maximum vertical height loss of the occlusal part of the restoration. To measure the wear, a region of interest (see Fig 3) was defined within the filling material in the area of the marginal ridge that was subjected to mechanical stress. The maximum vertical height loss was then measured within this region of interest.

Statistical analysis was carried out using the software program “IBM® SPSS® Statistics 26.0” (IBM, Armonk, New York, USA). Data were checked for variance homogeneity and normal distribution (prerequisite test). ANOVA was used for the inductive statistics. Non-parametric tests, such as the Kruskal–Wallis test for non-connected samples for inter-material related multiple group comparisons, the Mann–Whitney U test for non-connected samples for dentition-related single group comparisons and the Wilcoxon signed-rank test for connected samples, were used in the event of a violation of the preconditions. The results of the multiple group comparisons were corrected for the parametric tests using the Sidak test and for the non-parametric tests using the Bonferroni–Holm method to avoid an alpha error. Marginal criteria, that did not show a median value of ≥15% in any of the material groups, were excluded from the inductive statistical analysis.

A significance level (α) of 5 % was set for the entire statistical analysis. The median, the 25% quartile and the 75% quartile were used as representative measures for the descriptive statistics.

## RESULTS

### Margin Analysis

A median value of ≥15% was detected for the criteria “perfect margin,” “positive step formation,” “negative step formation,” and “gap.” The criterion “perfect margin” before and after thermomechanical loading (TML) and the criterion “positive step formation” after TML met the requirements for the use of parametric tests. An overview of the descriptive values of the margin analysis criteria that were included into inductive statistics is depicted in Table 2.

**Table 2:** Descriptive values for criteria included into inductive statistics and comparisons of the dentitions with regard to the different margin analysis criteria

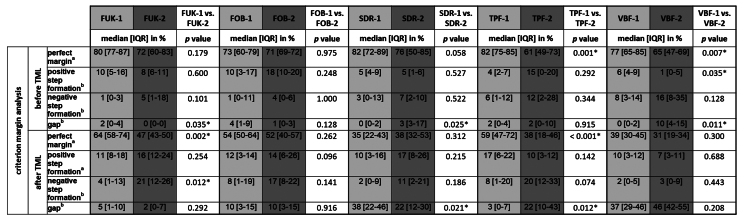

#### Comparisons of marginal characteristics within material groups before versus after TML (Wilcoxon signed-rank test)

**Fig 4:**
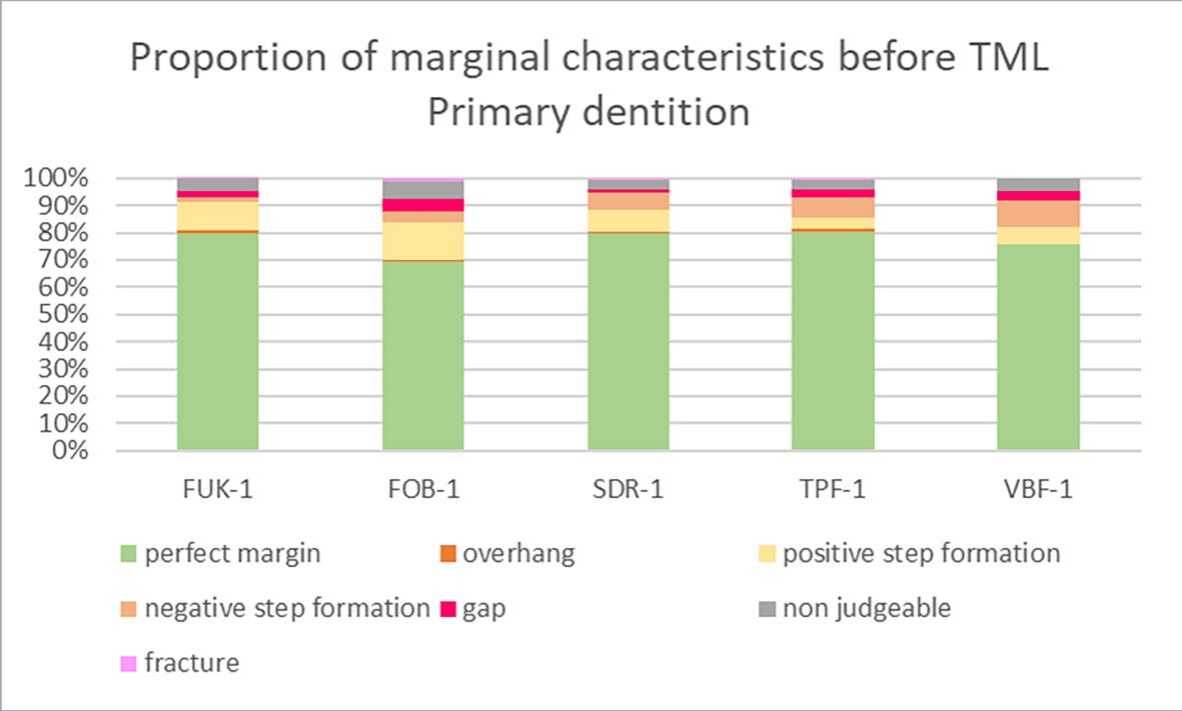
Stacked column chart showing the proportion (%) of the marginal characteristics of the different materials before thermomechanical loading (TML) for the tooth samples of the primary dentition. Abbreviations of the different materials are explained in Table 1.

**Fig 5:**
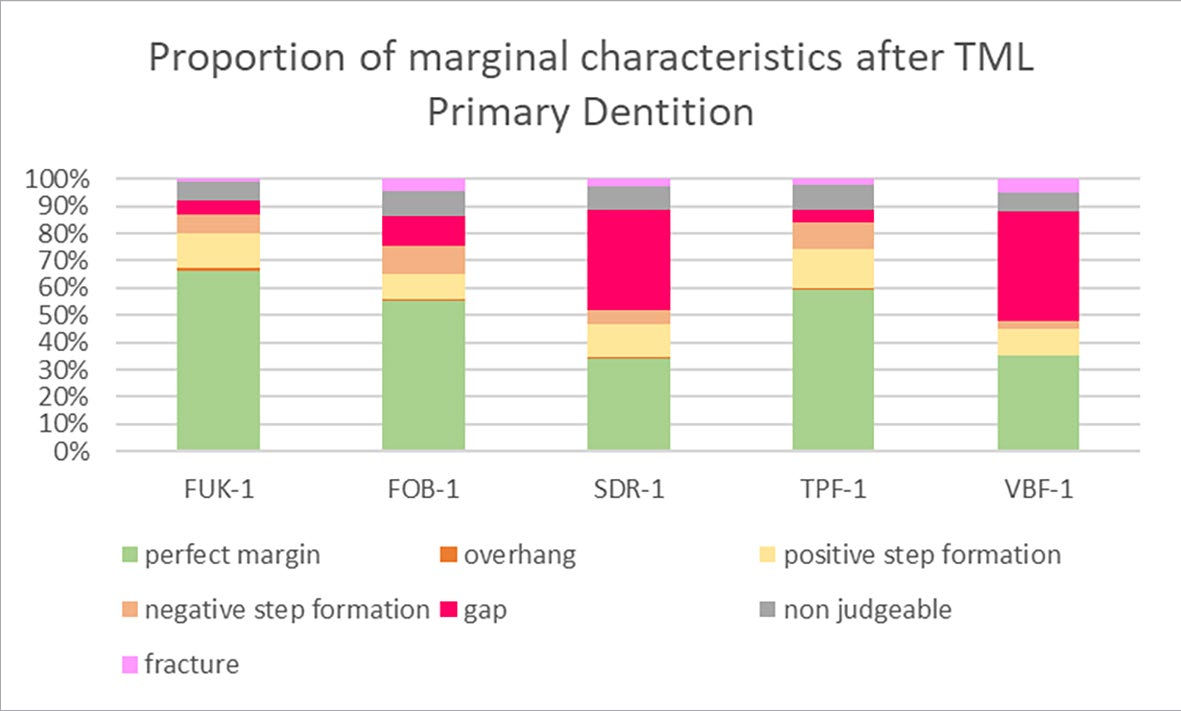
Stacked column chart showing the proportion (%) of the marginal characteristics of the different materials after thermomechanical loading (TML) for the tooth samples of the primary dentition. Abbreviations of the different materials are explained in Table 1.

**Fig 6:**
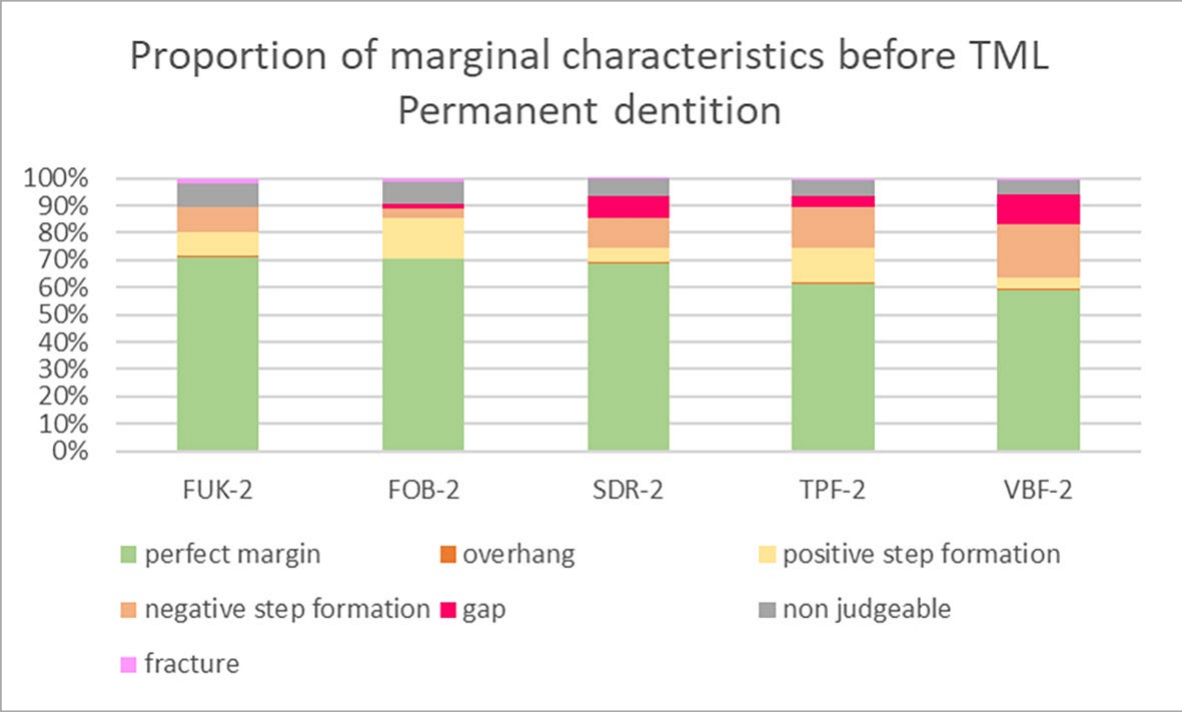
Stacked column chart showing the proportion (%) of the marginal characteristics of the different materials before thermomechanical loading (TML) for the tooth samples of the permanent dentition. Abbreviations of the different materials are explained in Table 1.

**Fig 7:**
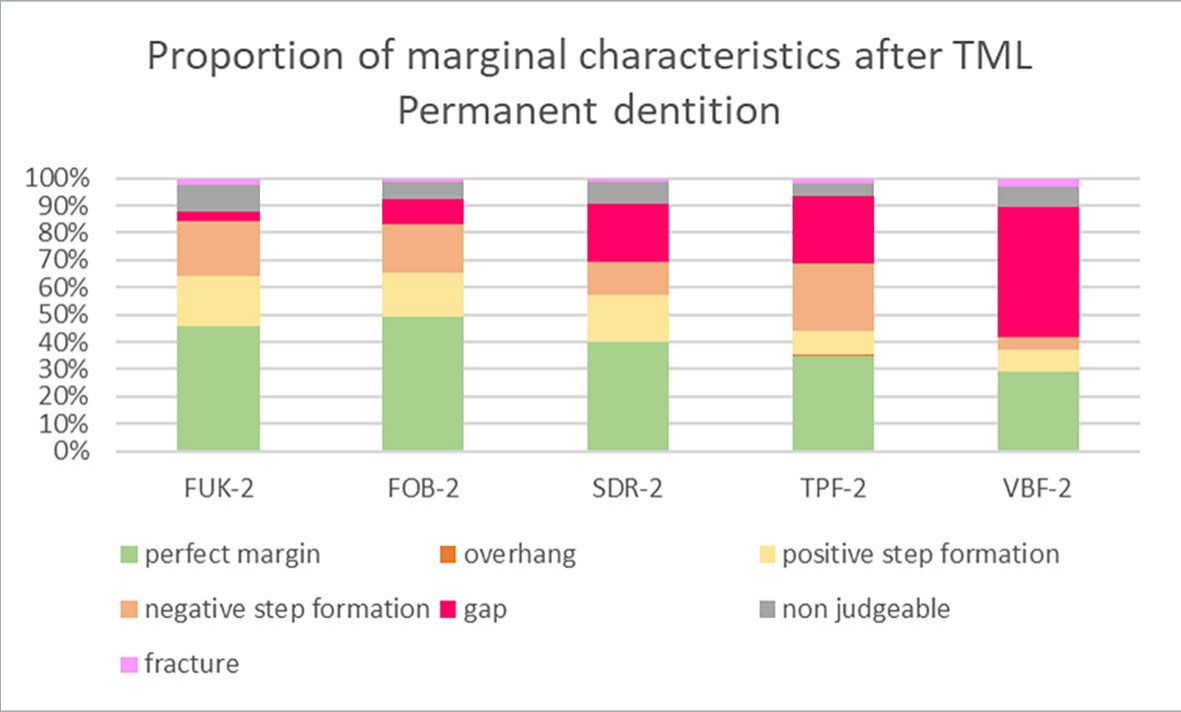
Stacked column chart showing the proportion (%) of the marginal characteristics of the different materials after thermomechanical loading (TML) for the tooth samples of the permanent dentition. Abbreviations of the different materials are explained in Table 1.

For both dentitions, a significant reduction of perfect margin areas could be observed after TML (P < .02). Positive step formations were detected significantly more often after TML than before TML for TPF-1 (P < .03), SDR-2 (P < .02), and FUK-2 (P < .02). Negative step formations occurred significantly more often for FUK-2 (P < .02) and for FOB-2 (P < .03), and significantly less often for VBF-2 (P < .03) after TML. Marginal gaps could be detected significantly more often after TML for FUK-1 (P < .03), SDR-1 (P < .02), VBF-1 (P < .02), FUK-2 (P < .02), FOB-2 (P < .02), SDR-2 (P < .02), TPF-2 (P < .02), and VBF-2 (P < .02) (Figs 4 to 7).

#### Material group comparisons within the dentitions (ANOVA/Kruskal–Wallis test)

**Fig 8:**
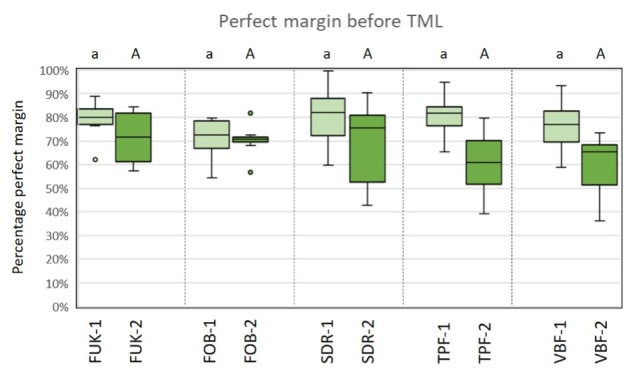
Box-plot diagram of the results of the margin analysis for the criterion “perfect margin” before thermomechanical loading (TML). Material groups of the primary dentition are marked in light green and material groups of the permanent dentition are marked in dark green. Abbreviations of the different materials are explained in Table 1. Different capital (permanent dentition)/lowercase (primary dentition) letters show significant differences (ANOVA, p<0.05) between groups.

**Fig 9:**
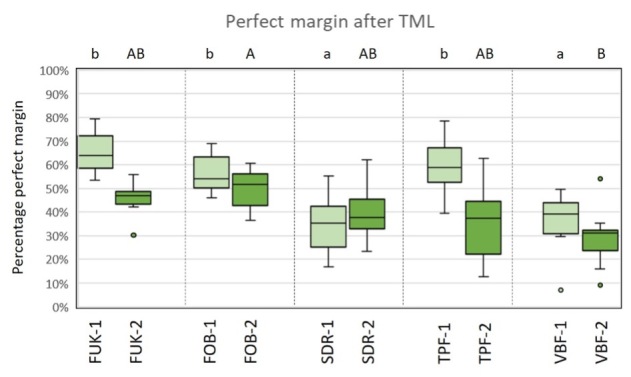
Box-plot diagram of the results of the margin analysis for the criterion “perfect margin” after thermomechanical loading (TML). Material groups of the primary dentition are marked in light green and material groups of the permanent dentition are marked in dark green. Abbreviations of the different materials are explained in Table 1. Different capital (permanent dentition)/lowercase (primary dentition) letters show significant differences (ANOVA, P < .05) between groups.

For material group comparisons within the dentitions, no significant differences for the criterion “perfect margin” before TML could be observed (ANOVA, P > 0.05). After TML, SDR-1/VBF-1 showed significantly less perfect margin areas than FUK-1 (ANOVA, P < .001/P < .001), FOB-1 (ANOVA, P < .005/ P < .001) and TPF-1 (ANOVA, P < .001/P < .002) for the groups of the primary dentition. Furthermore, FOB-2 showed a significantly higher number of perfect margin areas than VBF-2 (ANOVA, P < .02) for the groups of permanent dentition. A graphic representation of the results of the group comparisons for the criterion “perfect margin” before and after TML is depicted in Figures 8 and 9.

Before TML, no significant differences between groups of the primary dentition could be observed for the criterion “positive step formation” (Kruskal–Wallis test, P > 0.05). For the groups of permanent dentition, a significantly higher number of positive step formations could be observed for FOB-2 in comparison to VBF-2 (Kruskal–Wallis test, P < .03). After TML, no significant differences for the criterion “positive step formation” could be observed between the groups of both dentitions (ANOVA, P > 0.05).

For the criterion “negative step formation,” no significant differences between groups could be detected before TML for both dentitions and after TML for the primary dentition. For the groups of the permanent dentition after TML, VBF-2 showed significantly fewer negative step formations than TPF-2 (Kruskal–Wallis test, P < .02) and FUK-2 (Kruskal–Wallis test, P < .03).

Regarding the criterion “gap,” there were no significant differences between the groups of the primary dentition before TML (Kruskal–Wallis test, P > 0.05). For the permanent dentition, there were significantly fewer marginal gaps detectable for FUK-2 than for SDR-2 (Kruskal–Wallis test, P < .02), and for VBF-2 (Kruskal–Wallis test, P < .001).

After TML, SDR-1/VBF-1 showed significantly more gaps than FUK-1 (Kruskal–Wallis test, P < .003/P < .002), FOB-1 (Kruskal–Wallis test, P < .05/P < .04) and TPF-1 (Kruskal–Wallis test, P < .002/ P < .001). For the permanent dentition, VBF-2 showed significantly more marginal gaps than FUK-2 (Kruskal–Wallis test, P < .001) and FOB-2 (Kruskal–Wallis test, P < .005), and SDR-2 showed significantly more marginal gaps than FUK-2 (Kruskal–Wallis test, P < .04) after TML.

**Fig 10:**
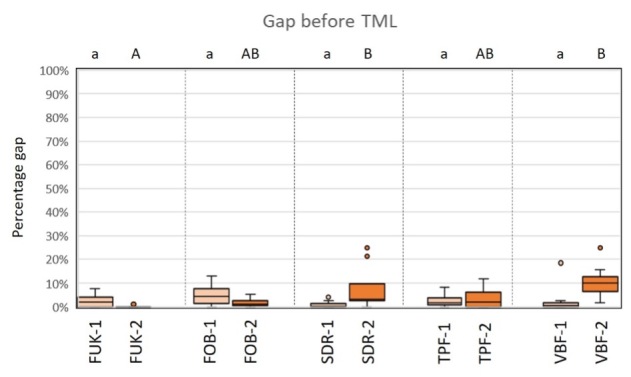
Box-plot diagram of the results of the margin analysis for the criterion “gap” before thermomechanical loading (TML). Material groups of the primary dentition are marked in light orange and material groups of the permanent dentition are marked in dark orange. Abbreviations of the different materials are explained in Table 1. Different capital (permanent dentition)/lowercase (primary dentition) letters show significant differences (Kruskal–Wallis, P < .05) between groups.

**Fig 11:**
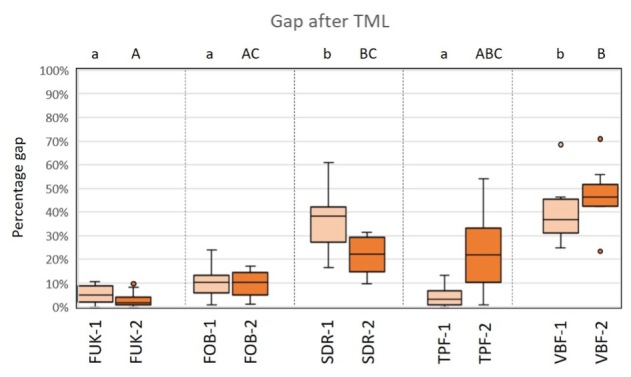
Box-plot diagram of the results of the margin analysis for the criterion “gap” after thermomechanical loading (TML). Material groups of the primary dentition are marked in light orange and material groups of the permanent dentition are marked in dark orange. Abbreviations of the different materials are explained in Table 1. Different capital (permanent dentition)/lowercase (primary dentition) letters show significant differences (Kruskal–Wallis, P < .05) between groups.

A graphic representation of the results of the group comparisons for the criterion “gap” before and after TML is shown in Figure 10 and Figure 11.

#### Comparisons of the dentitions (ANOVA/Mann–Whitney U test)

**Fig 12:**
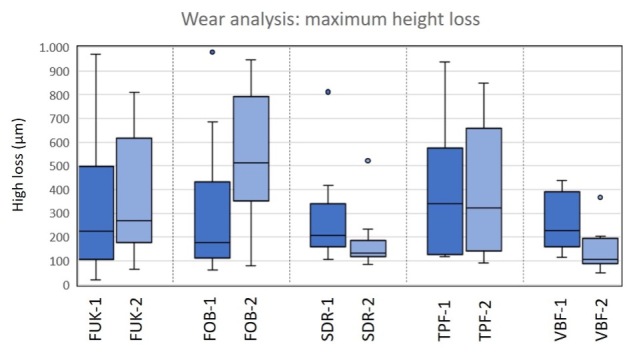
Box-plot diagram of the results of the wear analysis for the maximum height loss. There were no significant differences between material groups within the dentitions (Kruskal–Wallis test, P > 0.05). Abbreviations of the different materials are explained in Table 1.

**Fig 13:**
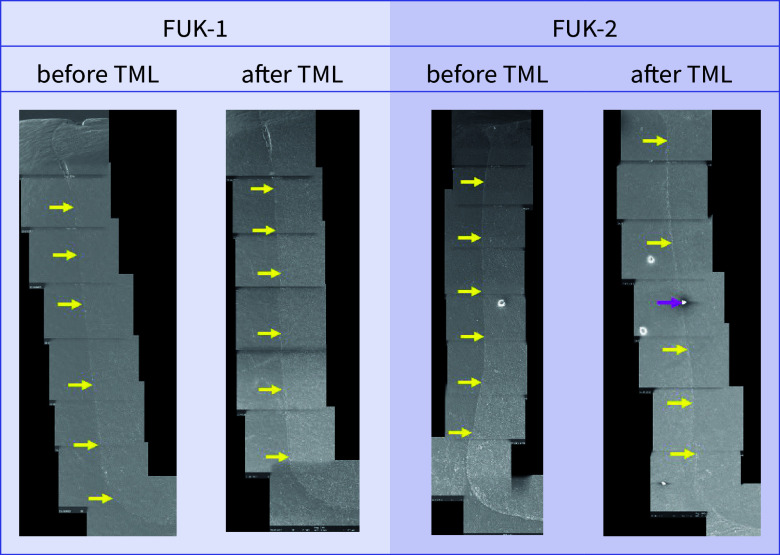
Exemplary scanning electron micrographs (200×) of the vertical filling margin area. Arrows are localized on the side of the enamel. Yellow arrows = perfect margin. Pink arrows = artificial particle deposits in the filling margin area. TML = thermomechanical loading. Abbreviations of the groups can be found in Table 1.

**Fig 14:**
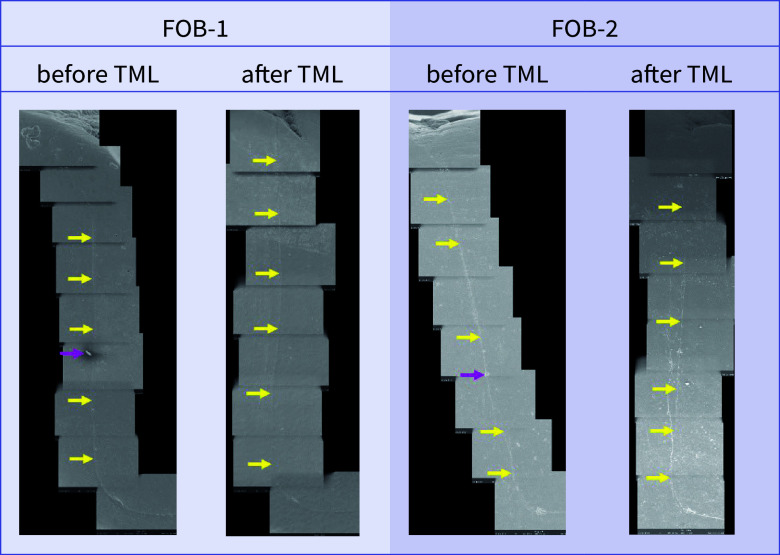
Exemplary scanning electron micrographs (200×) of the vertical filling margin area. Arrows are localized on the side of the enamel. Yellow arrows = perfect margin. Pink arrows = artificial particle deposits in the filling margin area. TML = thermomechanical loading. Abbreviations of the groups can be found in Table 1.

**Fig 15:**
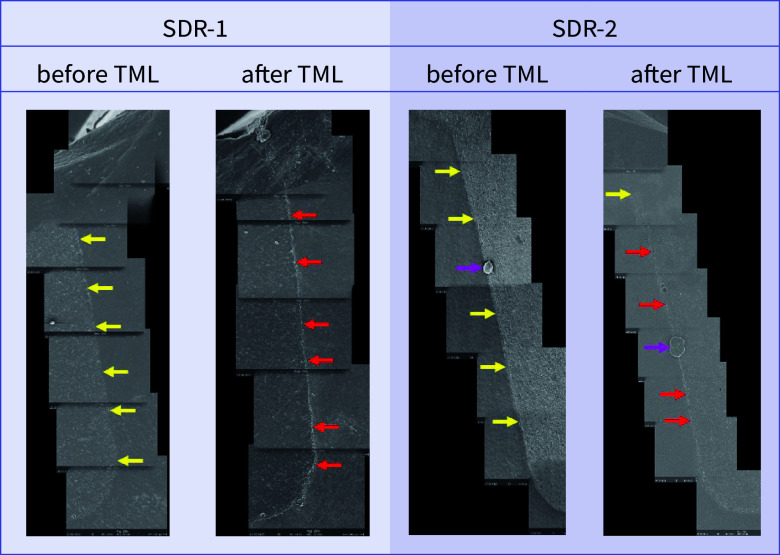
Exemplary scanning electron micrographs (200×) of the vertical filling margin area. Arrows are localized on the side of the enamel. Yellow arrows = perfect margin. Red arrows = gap. Pink arrows = artificial particle deposits in the filling margin area. TML = thermomechanical loading. Abbreviations of the groups can be found in Table 1.

**Fig 16:**
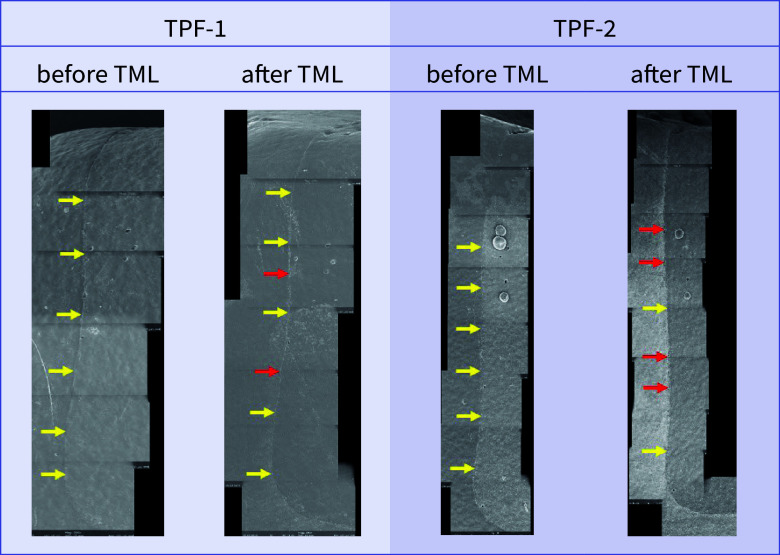
Exemplary scanning electron micrographs (200×) of the vertical filling margin area. Arrows are localized on the side of the enamel. Yellow arrows = perfect margin. Red arrows = gap. TML = thermomechanical loading. Abbreviations of the groups can be found in Table 1.

**Fig 17:**
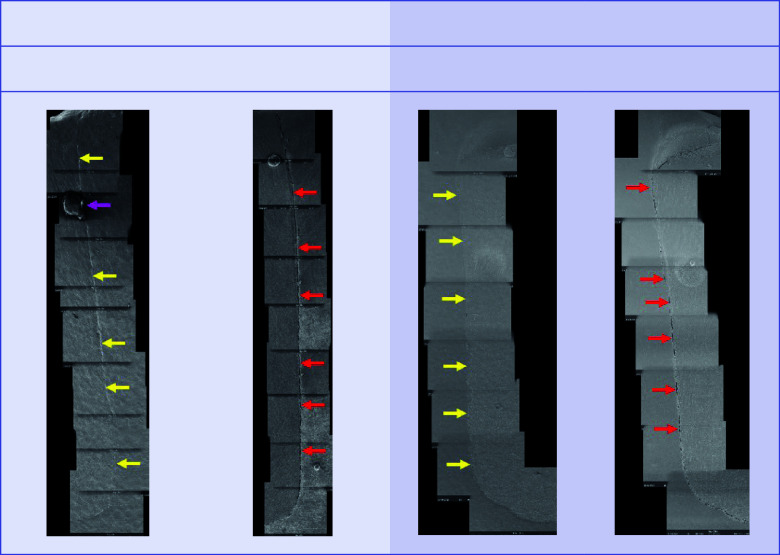
Exemplary scanning electron micrographs (200×) of the vertical filling margin area. Arrows are localized on the side of the enamel. Yellow arrows = perfect margin. Red arrows = gap. Pink arrows = artificial particle deposits in the filling margin area. TML = thermomechanical loading. Abbreviations of the groups can be found in Table 1.

Before and after TML, there were several significant differences between the samples from different dentitions that shared the same filling material. An overview of the comparisons of the dentitions with respect to each margin analysis criterion that was included in the inductive statistics is depicted in Table 2. For each material, exemplary scanning electron micrographs of both dentitions before and after TML can be found in Figures 10 to 17.

### Wear Analysis

#### Material group comparisons (Kruskal–Wallis test)

Within the two dentitions, no significant differences between material groups with regard to maximum height loss in the occlusal contact area could be observed (P < .05). A graphical representation of the distribution of the values for the maximum height loss is shown in Figure 12.

#### Comparisons of the dentitions (Mann–Whitney U test)

Significant differences between the dentitions could only be found for the material “Venus® Bulk Flow ONE” (VBF-1 versus VBF-2; P < .05). The descriptive values of the maximum height loss of each material group and the results of the comparisons of the dentitions of the wear analysis can be found in Table 3.

**Table 3:** Descriptive values (maximum height loss) of wear analysis and comparisons of the dentitions.

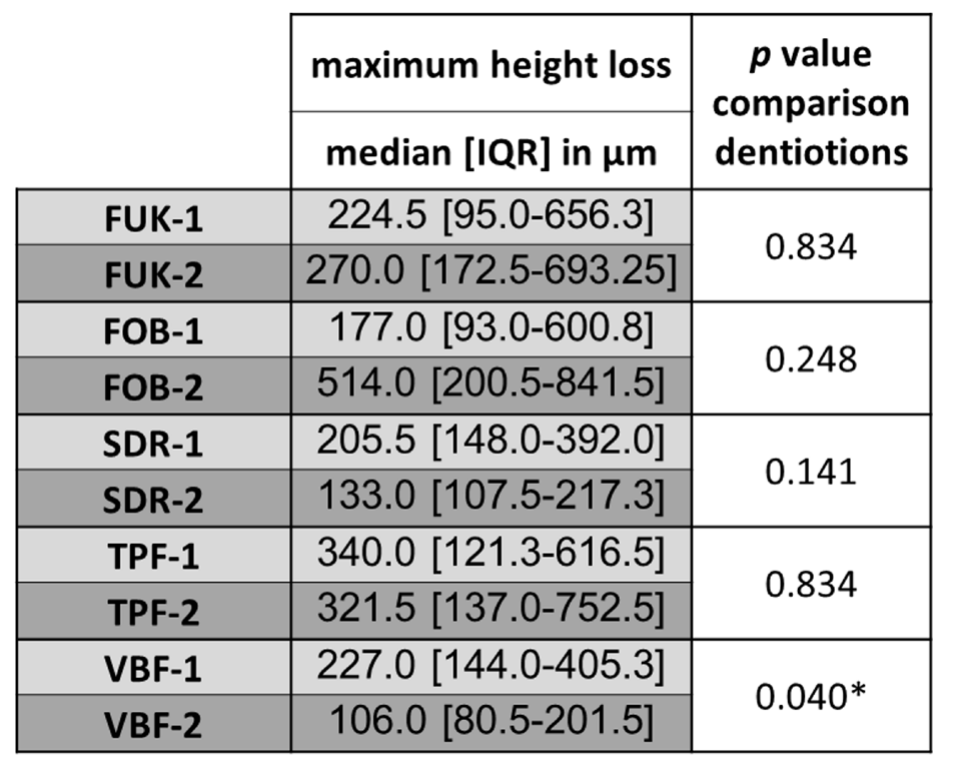

## DISCUSSION

Sufficient polymerization of the adhesive and the composite resin material is a key factor for successful restorative therapy. It is recommended to stick to a curing time of at least 20 s for composite resins using high-power LED polymerization lights,^29^ as was done in this study. Current studies also show that 20 s polymerization time should be adhered to for BFs. For certain bulk-fill composite products, a shorter polymerization time is not recommended due to an insufficient polymerization depth.^11,16,45^ For the adhesive system, the manufacturer’s instructions of 10 s polymerization time were implemented uniformly for all groups.

When comparing similar restorative material examinations with procedures for thermomechanical loading, it is noticeable that the number of thermocycling cycles and chewing simulation cycles often varies.^7,19,20^ Therefore, when analyzing the results, the focus should be on the relationships between the tested materials and not on the absolute values. The number of cycles used in this study have proven to be reliable in the context of multiple restorative material tests for both dentitions.^21,37,46^

With regard to the margin analysis before TML (T1), the criteria “perfect margin” and “gap” were most important for the interpretation of the marginal integrity of the filling because positive and negative step formations could be explained by too little or too much finishing and polishing, or they could be due to volumetric changes in the filling material during the storage in water.^22^ Initially, before TML, detected gaps could be interpreted as a result of the polymerization shrinkage of the composite material.^12^ Margin analysis revealed no significant differences between the material groups with regard to perfect margin areas at T1. Overall, there were only a few significant differences for a few evaluation criteria at the baseline. It can therefore be assumed that the groups can be compared well at T1.

After TML, gaps may have remained or have resulted from the degradation of the adhesive bond due to artificial aging.^6^ Regarding perfect/gap-free margins and marginal gaps, the high-viscosity bulk-fill composites tested in this study showed results comparable to an incrementally applied RC. These findings are consistent with multiple results in the literature, both for the primary and for the permanent dentition.^7,17–19,38,39^ Furthermore, the results of this study showed a superiority of the highly viscous BFs as well as RCs compared to the flowable BFs in terms of marginal integrity after TML, which is also confirmed by the results of other *in-vitro* studies with similar study designs for both dentitions.^20,44^ 3M™ Filtek™ One Bulk Fill showed good clinical outcomes in permanent teeth after one year of evaluation in a clinical trial by Cieplik et al (2022), where marginal adaption and wear according to the FDI criteria were also examined.^10^ For the other high viscous BF, Tetric® PowerFill, there are currently no clinical data available, but Tetric® PowerFill showed results comparable to other packable and flowable BFs as class V restorations in terms of microleakages in an *in-vitro* study.^30^

For samples of the primary dentition, both low-viscosity bulk-fill materials, SDR® flow+ and Venus® Bulk Flow ONE, showed significantly fewer continuous margins and significantly more gaps after TML than the packable BFs 3M™ Filtek™ One Bulk Fill and Tetric® PowerFill, and the packable RC, 3M™ Filtek™ Universal Komposit Z250 (see Figure 9 and Figure 11).

In the literature of the field, flowable bulk-fill composites were reported to show higher polymerization shrinkage due to their lower filler content.^13,27^ Gaps at the filling margin before TML, which could have been explained by polymerization shrinkage, could not be detected at a significantly higher level for the two flowable BFs in this study. However, the stress caused by the polymerization shrinkage in the restorative material could have further increased the formation of marginal gaps due to the TML in the low-viscosity groups of the primary tooth samples. In contrast, the flowable bulk-fill composite SDR® flow has clinically proven itself in the primary dentition in a split-mouth design study with results comparable to a conventional composite, with the assessment of the marginal integrity as one of the evaluated factors.^47^ Currently, no clinical data are available for Venus® Bulk Flow ONE or the two tested high viscous BFs as filling materials for primary molars. Since Venus® Bulk Flow ONE showed comparable results to SDR® flow here, a similar clinical behavior could be assumed. Compared to its results in the primary dentition, however, SDR® flow showed a different behavior in the permanent dentition after TML compared to Venus® Bulk Flow ONE. According to the descriptive data, SDR-2 showed fewer marginal gaps than VBF-2 and a comparable number of gaps when compared to the two highly viscous BFs after TML. It can therefore be assumed that SDR® flow works better in combination with the etch-and-rinse technique and the adhesive used on the tooth structure of the permanent teeth. This assumption is supported by a study by Al-Harbi et al (2015), in which the microtensile bond strength of the bond between SDR and the tooth structure of permanent teeth could be increased by the additional use of phosphoric acid in the adhesive protocol.^3^

Van Dijken and Pallesen (2015) published a 3-year follow-up where SDR performed well in combination with a capping layer for Classes I and II cavities in permanent molars, eg, for anatomical form, marginal adaption, surface roughness, and secondary caries. Furthermore, a comparable annual failure rate to a RC could be observed, even when using SDR in combination with a self-etch and not an etch-and-rinse technique.^49^

Differences between the dentitions within two groups that shared the same filling material were only found for a few evaluation criteria. These mainly concerned the criteria “perfect margin” and “gap.” 3M™ Filtek™ One Bulk Fill was the only material for which no significant differences were found between the first and second dentition. This result is consistent with the results of a study by Hamza et al (2022), which also showed no differences in marginal integrity between deciduous and permanent teeth for the same bulk-fill material.^20^ Before TML, Tetric® PowerFill and Venus® Bulk Flow ONE showed significantly more gap-free marginal areas for the primary dentition than for the permanent dentition. This result is also consistent with the study by Hamza et al (2022), where all materials in the primary dentition showed better marginal integrity before TML.^20^ However, this could not be proven for the other three materials in this study. SDR® flow+ showed controversial results in the dentition comparison for the “gap” criterion before and after TML. Before TML, there were significantly fewer marginal gaps on the primary tooth than on the permanent tooth, and vice versa after TML. This could be interpreted to mean that the self-etch technique with the adhesive system used initially withstands the polymerization shrinkage in the primary dentition better than the etch-and-rinse technique of the same system in the permanent dentition. Subsequently, the etch-and-rinse technique appears to be superior to age-related (simulated) stress within the restorative material or the adhesive bond.

Al Sheikh (2022) examined the volume of the marginal gaps of different BFs and RCs, and reported the lowest gap volume for SDR® flow+ and the highest gap volume for 3M™ Filtek™ One Bulk Fill. Furthermore, a generally smaller gap volume in dentin areas for BFs in comparison with RCs was detected.^4^ It should therefore be considered whether it is not the quantity of gaps that occur, but also their size that is of particular importance for bacterial penetration, and, if marginal gaps in dentin and not in enamel are the key factor for the occurrence of recurrent caries.

The evaluation of the wear analysis did not reveal any significant differences between the individual material groups within the dentitions. This result is consistent with a previous study, in which the wear of the same bulk-fill composites (apart from Venus® Bulk Flow ONE) as in the present study was examined *in vitro* on primary teeth.^21^ There were also no significant differences in terms of wear compared to a compomer material. Since compomers have long been used for the filling of primary molars, Hofmann et al concluded that bulk-fill composites are an adequate alternative filling material for primary dentition in terms of wear resistance.^21^ This conclusion can be applied to the results of this study. Furthermore, when comparing the same material in primary and permanent teeth, a significant difference regarding wear resistance could only be observed for Venus® Bulk Flow ONE, having shown a higher maximum height loss for the primary tooth samples. Since wear resistance of the tooth structure can influence the wear results when testing the wear of a filling material within a natural tooth sample, this result could be due to parallel antagonist contact areas in the tooth structure, which is less wear-resistant for primary teeth.^25^ Based on the results of the wear analysis in this study, it seems questionable whether all bulk-fill composites need to be covered with a RC, or whether this should be decided on an individual basis depending on the restorative material and the type of tooth structure.

To date, only a few reports have been published dealing with BFs *in vitro* for both primary and permanent dentition under such standardized conditions. The *in-vitro* studies published to date, which examine BFs for both dentitions, investigated marginal integrity/microleakage, microhardness, flexure strength, elastic modulus, and surface roughness.^20,23^ In only one of the studies TML, ie, a combination of mechanical and thermal fatigue stresses was used.^20^ Moreover, none of the studies published to date have investigated the wear behavior of BFs in the permanent dentition (and especially without a capping layer) in comparison to the primary dentition. Furthermore, there is no clinical study that has examined the performance of BFs for both primary and permanent teeth.

3M™ Filtek™ Universal Z250 showed good results *in vitro* with regard to both parameters analyzed in this study. However, it should be considered that RCs in clinical use require a higher number of steps with rather high technique sensitivity,^15^ including the absence of contamination. This cannot be implemented for all patient groups, especially not for young children. Therefore, bulk-fill composites offer a good restoration material alternative, particularly if time is the limiting factor of the restoration procedure. Since it cannot be predicted with certainty that imperfections or gaps in the restoration margin will indeed result in the formation of secondary caries,^41^ there is a need for further research, particularly clinical research. Future clinical studies should examine the long-term success of the various restorative materials for both dentitions in order to verify the transferability of the research results to the clinical situation. More clinical data would also be beneficial with regard to wear analysis, particularly for the primary dentition where clinical data are rare, for example by using intraoral scanners to document wear in the oral cavity. Future studies should further examine the extent to which marginal quality can be influenced or improved by optimizing the matching of restorative material and adhesive system or adhesive technique.

Since BFs show satisfactory results in both dentitions, it could be assumed that BFs represent a future “universal filling material” for both dentitions. This would eliminate the need to purchase different filling materials for different tooth substrates when treating both children and adults.

In conclusion, all of the tested bulk-fill composite resins showed satisfactory *in-vitro* results for both tested parameters in primary and permanent teeth with a superiority of the high-viscosity materials in terms of marginal integrity and should be clinically evaluated.

### Clinical Relevance

The micromorphological differences between teeth of the primary and permanent dentition must be considered in restorative therapy with bulk-fill composites, but the individual compatibility of the restorative material and adhesive system should also not be disregarded.
